# Childhood-onset systemic lupus erythematosus: characteristics and the prospect of glucocorticoid pulse therapy

**DOI:** 10.3389/fimmu.2023.1128754

**Published:** 2023-08-10

**Authors:** Lu Pan, Jinxiang Liu, Congcong Liu, Lishuang Guo, Marilynn Punaro, Sirui Yang

**Affiliations:** ^1^ Department of Pediatric Rheumatology, Immunology and Allergy, The First Hospital, Jilin University, Changchun, China; ^2^ Pediatric Rheumatology, University of Texas Southwestern Medical Center, Dallas, TX, United States; ^3^ Rheumatology, Texas Scottish Rite Hospital for Children, Houston, TX, United States; ^4^ Pediatric Rheumatology, Children’s Medical Center of Dallas, Dallas, TX, United States

**Keywords:** childhood-onset systemic lupus erythematosus, genetic factors, type I interferon, glucocorticoids, intravenous methylprednisolone pulse therapy

## Abstract

Childhood-onset systemic lupus erythematosus (cSLE) is an autoimmune disease that results in significant damage and often needs more aggressive treatment. Compared to adult-onset SLE, cSLE has a stronger genetic background and more prevalent elevated type I Interferon expression. The management of cSLE is more challenging because the disease itself and treatment can affect physical, psychological and emotional growth and development. High dose oral glucocorticoid (GC) has become the rule for treating moderate to severe cSLE activity. However, GC-related side effects and potential toxicities are problems that cannot be ignored. Recent studies have suggested that GC pulse therapy can achieve disease remission rapidly and reduce GC-related side effects with a reduction in oral prednisone doses. This article reviews characteristics, including pathogenesis and manifestations of cSLE, and summarized the existing evidence on GC therapy, especially on GC pulse therapy in cSLE, followed by our proposal for GC therapy according to the clinical effects and pathogenesis.

## 1 Introduction

Systemic lupus erythematosus (SLE) is a chronic autoimmune disease that affects every organ system and causes heterogeneous clinical manifestations (1). This disease mostly occurs in adults, especially females (2). Approximately 15% to 20% of all SLE patients are diagnosed during childhood (3). Although childhood-onset SLE (cSLE) has similar pathogenesis, clinical manifestations, and immunologic disorders to adult-onset SLE (aSLE), there are some differences between them (4). There is evidence that cSLE has stronger genetic background and Interferon (IFN) signature (5). Previous studies have shown that compared to aSLE, cSLE has a more aggressive course and frequent damage accrual, with high morbidity and mortality (6). Moreover, patients with cSLE generally require more aggressive treatment to achieve lupus low disease activity state (LLDAS) than aSLE patients (7). Indeed, the management of cSLE is more challenging because the disease itself and treatment can affect physical, psychological and emotional growth and development.

Glucocorticoid (GC) remain the cornerstone of treatment for SLE patients and are commonly used for prolonged periods, especially in children. Daily high-dose oral GC is the most common therapeutic management for moderate to severe cSLE patients during the induction period (8). However, the side effects caused by long-term use of medium to high doses of GC are unfortunate for children who are growing, and this profile has led to a search for an optimized GC therapeutic management that has the least side effects. Recently, the intravenous pulse form of GC seems to have greater advantages in SLE. GC pulse is the essential element for effectively treating active SLE using lower doses of oral GC. In addition, GC pulse is not associated with most side effects, which were only related with the occurrence of cognitive impairment or psychosis (9, 10). Combined with the characteristics of cSLE, the possibility of GC pulse to reduce GC-related side effects while maintaining the disease remission offers significant promise. Therefore, this review summarized the characteristics of cSLE and described important developments in GC, especially in GC pulse, to develop new ideas for GC therapeutic management of cSLE from the perspective of pathogenesis and clinical practice.

## 2 The characteristics of cSLE

### 2.1 The pathogenesis of cSLE

Similar to aSLE, cSLE pathogenesis is complex and not fully understood. However, increasing severity with younger age and varying gender distribution in different age groups suggest variable pathogenic mechanisms between aSLE and cSLE.

#### 2.1.1 Genetic factors

Familial clusters, the impact of ethnicity on disease prognosis, age-specific differences in clinical and immunological phenotypes, and the high degree of concordance among monozygotic twins suggest a stronger involvement of genetic factors in cSLE. A recent study that included multi-ethnic SLE patients suggested that there is a negative association between non-HLA genetic risk and age of SLE diagnosis (11). Compared to aSLE, cSLE patients are more likely to carry novel/rare high-penetrance variants associated with monogenic lupus or may have a burden of low-penetrance common SLE susceptibility alleles (12).

**Figure 1:**
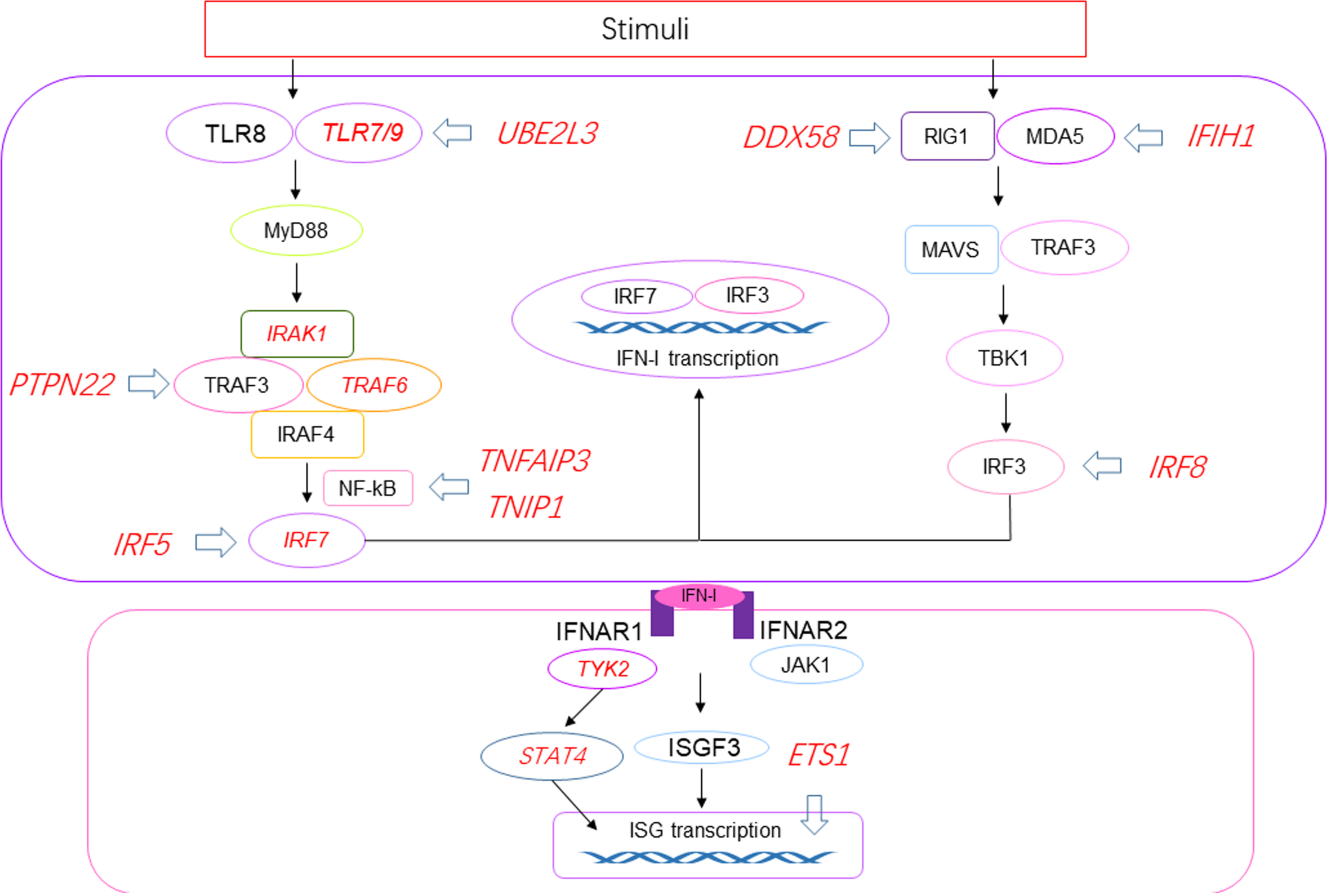
Susceptibility genes related to the production of IFN-I and IFN-I signaling pathway in SLE. Red italics represent susceptibility genes, and blue arrows represent proteins in the pathway affected by the susceptibility genes. UBE2L3 regulate the degradation of TLR4/9. High IFN-α in SLE patients is related to the risk allele of PTPN22 though TRAF3. TNFAIP3 and TNIP1 encode regulatory factors in IFN-I pathway, NF-κB activation. DDX58 is related to RIG1 hyperactivation. IFIH1 encodes MDA5 and is involved in elevated expression of IFN-induced genes. ETS1 is a negative regulator in SLE. IRF5/7/8 can directly induce transcription of proteins in IFN-I signaling pathway. IRAK1, TRAF6, STAT4 and TYK2 are related to the production of IFN-I. UBE2L3, ubiquitin-conjugating enzyme E2 L3; PTPN22, protein tyrosine phosphatase non-receptor type 22.

Recently, cSLE has been found to be associated with single gene mutations, defining the concept of monogenic lupus. Genes linked to monogenic lupus belong to type I interferonopathies, the complement deficiencies, T and B cell tolerance breakdown, or other uncharacterized pathways (13).First, type I interferonopathies refer to a group of complex genetic disorders associated with imbalance of IFN mediated immune responses. The mutations identified so far cause IFN overexpression in three different pathways: defect in nucleases (TREX1, SAMHD1, ADAR1, RNASEH2), enhanced sensitivity of an innate immune sensor (IFIH, DDX58) or adaptor molecules downstream the innate sensors (TMEM173), and defective negative feedback of the IFN pathway (ISG15) (14). Furthermore, mutations in the ACP5 gene result in a deficiency of tartrate-resistant acid phosphatase (TRAP) enzyme, which eventually leads to excessive IFN production and the development of SLE (15). Mutations in genes involved in IFN signaling pathway are shown in **Figure 1**. Second, complement deficiency is another described subcategories of monogenic lupus and highlight the importance of apoptotic body clearance in lupus pathogenesis (16). Genetic deficiencies of C1q/r/s strongly predispose to SLE, with a penetrance of nearly 90% (17). Deficiencies in other complement component also promote SLE, but the risk is lower. Notably, complement deficiencies are associated with early-onset lupus, and has a less biased of sex ratio compared to aSLE. Third, tolerance breakdown caused by genetic mutations in B and/or T cell promotes SLE. Protein kinase C-δ (PKC-δ) is a serine/threonine kinase important in multiple apoptotic signaling cascades. Mutation in PKC-δ is associated with loss of B cell tolerance and has been identified in cSLE patients (18). Deficiency in RAG1/RAG2 lead to defects in T cell tolerance, and a patient with SLE was reported to have heterozygous mutation in RAG2 recently (19). Other genes involved in monogenic lupus including DNASE1/DNASE1L3 encoding proteins involved in the nucleic acid degradation pathway and CYBB gene causing chronic granulomatous disease.

Indeed, single locus confers only a small disease risk. Most individuals carry risk alleles and develop SLE with the influence of environment and time. Genome-wide association studies (GWAS) have identified more than 100 risk loci, accelerating the discovery of common single nucleotide polymorphisms (SNPs) (20). Compared with aSLE, cSLE are less affected by environment and time, but have more severe clinical manifestations, which may be related to increased genetic risk. The calculation of genetic risk score (GRS) by counting the number of SLE-associated risk alleles weighted odds ratios (ORs) has more power to predict disease susceptibility (21). Studies in Gullah and African-Americans showed an increased number of SLE-associated polymorphisms in cSLE compared with aSLE (22). Similarly, a Korean study showed that cSLE had a higher genetic risk scores (GRS) than aSLE (23). A recent study showed that there was a trend for stronger associations between both GRS and LN risk in Europeans with cSLE compared with aSLE (24). The evidence for common variants being directly pathogenic is limited. Next generation sequencing (NGS) technologies, including whole genome sequencing (WGS) and whole exome sequencing (WES), have led to increasing recognition that rare/novel variants are more deleterious than common variants (25). A previous study explored rare variants by undertaking WES of SLE patients showed that 14 missense de-novo variants were identified in SLE probands (26). In recent years, more and more rare variants that cause SLE have been identified, such as novel peripheral gene MEF2D, germline rare P2RY8 missense variants and SAT1 LOF variants (27–29). A study in Mexicans showed that catalytically impaired TYK2 variants were protective against SLE, but there is no difference between aSLE and cSLE (30). The genetic variants in cSLE differed from those in aSLE are showed in **Table 1**.

**Table 1:** Different genetic variants between cSLE and aSLE.

Gene	Variant	Ethnicity	Genes with functional roles potentially relevant to SLE	Distinct between cSLE and aSLE	Ref
ESR1/ESR2	rs2234693 rs4986938	Polish	ESR1/ESR2 is involved in the pathogenesis of SLE through estrogen activation	ESR1 (rs2234693) was associated with cSLE. ESR2 (rs4986938) was associated with aSLE.	(31)
STAT4	rs7574865, rs7601754	Iranian	STAT4 may involve in SLE development by regulating Th1, Th17 and related cytokines.	The two SNPs of STAT4 have no relationship with the risk of cSLE, despite their association with the risk of aSLE in Iranian population	(32)
MECP2	rs1734787, rs1734791	Iranian	MECP2 encodes MeCP2, which changes the DNA methylation pattern, and then perturbate the epigenetic modifications of T cells to participate in SLE.	Although aSLE was associated with MECP2, this gene was not associated with disease susceptibility in cSLE patients	(33)
PDCD1	PD1.3A (rs11568821)	Mexican	PDCD1 contribute to the breakdown of preripheral tolerance to self-antigens and development of SLE	This SNP was associated with cSLE in Mexican, which was different from those reported aSLE in Spanish and Swedish.	(34)
ARID5B	rs10821936	Egyptian	The ARID5B protein plays an important role in the growth and differentiation of B-lymphocyte progenitors and possibly other lympocytes	This SNP was associated with cSLE, which has no association with all SLE in Chinese Han population	(35)
C4/C4B	Low gene copy number	Brazilian	C4 involved in the pathogenesis of SLE through the complement pathway	Low C4 gene copy number is a stronger risk factor for cSLE than aSLE. Low C4B gene copy number is associated with cSLE but not with aSLE.	(36)

STAT4, Signal transducer and activator of transcription 4; ESR, Oestrogen receptor; SNP, Single nucleotide polymorphisms; MECP2, Methyl-CpG-binding protein 2; PDCD1, Programmed cell deth 1; ARID, AT-rich interactive domain.

The clinical variability of SLE is further influenced by genetic modifiers, epigenetics and environmental factors. We will only describe the epigenetic mechanisms briefly. DNA methylation, posttranslational histone modifications, and non-coding RNAs are the main epigenetic mechanisms studied. DNA hypomethylation plays an important role in T cell activation and contributes to SLE pathogenesis (37). It is worth noting that some DNA methylation, such as long interspersed nuclear element-1 (LINE-1), differs between aSLE and cSLE (38). Abnormal histone acetylation and altered microRNA (miRNA) expression also play crucial roles in the pathological processes of SLE. Increased histone acetylation in monocytes and CD4^+^ T cells has been detected in SLE patients and inhibition of histone acetylation can alleviate disease activity (39). In recent years, there have been many studies on the progress of miRNA in SLE, such as miR-146a, miR-31 and miR-98 (40, 41). In addition, E2F transcription factor 2 (E2F2)-miR-17-5p was found to increase autoantibody production by upregulating IL-10 (42). Furthermore, miR-448 was involved in SLE by targeting the suppressor of cytokine signaling 5 (SOCS5) to promote helper T cell (Th)17 activation (43). Taken together, genetic factors play an important role in SLE, and the available data suggested that cSLE is more strongly influenced by genetic factors.

#### 2.1.2 Type I interferon

Type I IFN (IFN-I) is a multifunctional immune factor that bridges innate and adaptive immunity. It has been reported that IFN-I can promote the expansion of autoantibody-secreting cells (44) and more than half of the SLE-associated genetic loci are connected to the IFN-I pathway (45). Of note, the overexpression of IFN-regulated genes in the peripheral blood monocytes of almost all active cSLE patients was observed only in approximately 50% of aSLE patients (46, 47), revealing that cSLE is more closely related to IFN-I.

Plasmacytoid dendritic cells (pDCs) are the primary source of IFN-I. Under normal conditions, IFN-I production is strictly regulated for it ceases after pathogens have been cleared. However, many SLE patients demonstrate chronic overactivity in IFN-I pathways. Endogenous stimuli act upon a susceptible genetic background to result in IFN-I production.

In SLE, pDCs are activated by specific IFN immune complexes (ICs), derived from autoantibodies and endogenous or exogenous nucleic acid-binding proteins, and produce IFN-I (48). Interferogenics ICs are endocytosed through Fc gamma receptor IIa (FcγRIIa) on pDC and interact with TLR7 or TLR8/9 and initiate an activation chain that includes myeloid differentiation factor 88 (MyD88). MyD88 activates interleukin-1 receptor-associated kinase (IRAK) 4, then triggers IRAK1, tumor necrosis factor receptor associated factor (TRAF) 3 and TRAF6. Next, the interferon regulatory family (IRF) 7 is activated, resulting the initiate transcription of IFN-I (49). RIG-I and melanoma differentiation-associated gene 5 (MDA5) recognize ds-RNA and bind to mitochondrial antiviral signaling protein (MAVS), which activates TRAF3 and TANK-binding kinase 1 (TBK1) resulting the activation of IRF3 (50). Neutrophil extracellular traps (NETs) are another mechanism that triggers IFN-I generation. NETs accumulate in SLE patients, resulting in long-term exposure to the body and externalization of their antigens, thus producing high levels of IFN-I in a TLR9-dependent manner (51). In addition, mitochondrial reactive oxygen species (ROS) can initiate NETs formation (NETosis), activate the cyclic GMP-AMP synthase (cGAS)-stimulator of interferon genes (STING) pathway, and induce IFN-I production. IFN-I production further exerts its effects by ligating the IFN-α/β receptor (IFNAR). The Janus activating kinase (JAK)-STAT pathway is the most common signaling pathway. IFN-I combines with IFNAR to activate JAK1 and tyrosine kinase 2 (TYK2), and phosphorylated STAT1 and STAT2 form a transcription factor complex with IFN regulatory factor 9 (IRF9), which translocate to the nucleus, binds to IFN regulator elements in the promoters of IFN-regulated genes and initiates transcription genes (52). In SLE, IFN signaling-induced IFN-regulated genes participate in a positive feedback loop of autoimmunity, causing permanent autoimmune inflammation. Gene mutations related to IFN-I production or IFN-I signaling pathway (**Figure 1**) can lead to the production of self-derived IFN inducers and suppress the negative feedback signals that downregulate the IFN response.

**Figure 2:**
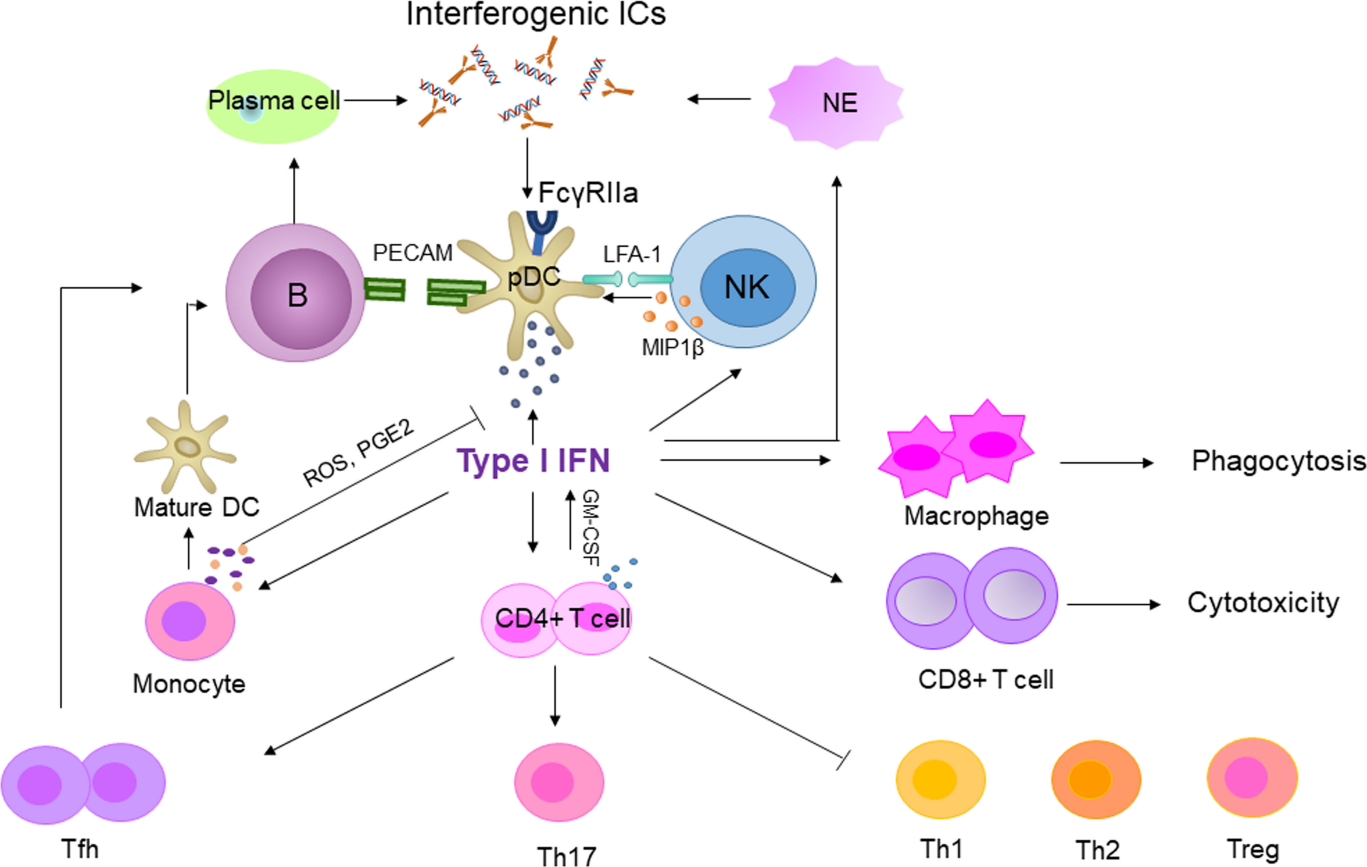
The central role of type I IFN in SLE. Interferogenic ICs stimulate pDCs and produce type I IFN. The secreted type I IFN acted on innate and adaptive immune cells to amplify the autoimmune response. B, T and NK enhanced type I IFN production by activated pDCs via PECAM, GM-CSF and LFA-1, respectively. In contrast, activated monocytes suppressed the secreted type I IFN by pDC via ROS and PGE2. GM-CSF, Granulocyte-macrophage colony stimulating factor; PGE2, Prostaglandin E2; NE, neutrophil.

IFN-I has a significant impact on the immune system. IFN-α promotes the expression of MHC-II and costimulatory molecules and stimulates monocytes to differentiate into mature dendritic cells (DCs) (53). Mature DCs and IFN-α activate B cells and increase autoantibody production. B cells interact with pDC through the platelet endothelial cell adhesion molecule (PECAM)-1 receptor to promote IFN-α secretion. IFN-α can also promote the differentiation of CD4^+^ T cells into Th1 and Th17, inhibit the development of Th2 and regulatory T cells (Treg), and enhance the cytotoxicity of CD8^+^ T cells. However, persistent IFN-α stimulation inhibits Th1 differentiation and promotes the development of T follicular helper (Tfh) cells, which supports B cell activation (54). Regarding the innate immune system, IFN-α enhances macrophage phagocytosis and natural killer (NK) cell cytotoxicity, and NK cells have a particularly strong effect on pDC through lymphocyte-associated antigen-1 (LFA-1)-dependent cell-cell interaction (55). Therefore, activation of the IFN-I system in SLE will widely affect the immune system (**Figure 2**), drive autoimmune response and chronic inflammation, and eventually cause tissue and organ damage. What’s more, children with SLE had elevated TLRs expression, and TLRs can induce neutrophil apoptosis, which lends more support to the role of IFN-I in cSLE (56).

### 2.2 The manifestations of cSLE

aSLE and cSLE differ in some disease phenotypes. Children have less gender bias in favor of females than adults. Moreover, children have higher SLE disease activity index (SLEDAI) scores than adults, and the earlier the age of onset, the greater the disease activity (57). This heterogeneity is broader in terms of clinical manifestations.

Systemic presentations, such as neurological, renal, and haematological involvement, are more significant with cSLE, whereas Raynaud’s phenomenon, pulmonary involvement, and photosensitivity are more common with aSLE (58) (**Table 2**). Among these, nephritis is the most important variation. A previous study showed that crescents on biopsy were more common in the cSLE (69). Another study reported the proportion of adults and children with lupus nephritis receiving kidney transplantation to be 1.9% and 3%, respectively (70). Neurological involvement is another important variation. A recent meta-analysis of neuropsychiatric symptoms in cSLE revealed that neuropsychiatric events were common in cSLE, including headaches, cognitive dysfunction, mood disorders and psychosis (71). Childhood-onset lupus remains a strong predictor of mortality. Serologically, cSLE and aSLE have similar positivity rates for most circulating antinuclear antibodies. However, a recent study showed that children had more anti-double-stranded DNA (dsDNA) and anticardiolipin immunoglobulin M (IgM) but less anti–Sjögren’s-syndrome-related antigen A (anti-Ro) and anti–Sjögren’s-syndrome-related antigen B (anti-La) antibodies (62). Antiphospholipid antibody-related thrombosis is uncommon in children but common in adults (72). In addition, rheumatoid factor positivity is more frequently encountered in aSLE, whereas arthritis is more common in cSLE (4).

**Table 2:** Clinical and immunological characteristics in cSLE and aSLE.

Characteristics	cSLE	aSLE	Ref.
**Gender, female/male**	2.7:1-6.5:1	8.4:1-16.7:1	(59, 60)
**Age at diagnosis (years, mean)**	12.7-15.5	28.3-37.0	(4, 58)
**Prevalence of SLE**	1-6/100,000	20-70/100,000	(61)
**Percentage of SLE (%)**	10%-20%	70%-87.4%	(62)
Clinical manifestations (%)			
**Systemic** Fever Lymphadenopathy	20.4%-68.4% 6.9%-29.6%	15.4%-64.9% 4%-19.3%	(63, 64) (65)
**Mucocutaneous** Malar rash Discoid rash Photosensitivity Oral ulcers	22.4%-71.9% 0%-26.5% 33.5%-75% 5.3%-40.8%	16.7%-61% 3.5%-14.9% 43.5%-72.5% 6.7%-48.5%	(60, 66) (62, 63) (62, 65) (4, 62)
**Arthritis**	39.2%-85%	22.4%-84.9%	(59)
**Serositis** Pericarditis Pleurisy	5%-24.8% 1.7%-24.8%	2.7%-16% 2.5%-16.5%	(4, 6) (4, 6)
**Neuropsychiatric**	9.9%-36.7%	6%-20%	(4, 67)
**Hematologic disorders** Hemolytic anemia Leucopenia/lymphopenia Thrombocytopenia	9.3%-38.6% 11.1%-61.7% 15%-52.6%	9.13%-24.5% 8.6%-56.9% 15.5%-39.6%	(6, 60, 62) (59, 64) (6, 63)
**Nephritis**	42.7%-83%	27.1%-67%	(68)
**Thrombosis**	2.7%-4%	12%-15.4%	(4, 65)
Autoantibodies (%)			
Anti-dsDNA	41.2%-89.7%	37.9%-88%	(59, 63)
Anti-Smith	16.3%-30.3%	10%-45%	(58, 62)
Anti-RNP	0.7%-28%	1.8%-33.6%	(4, 60, 62)
aCL	2%-37%	4.6%-24.1%	(6, 60, 62)
**SLEDAI, mean**	4.4-20	4.6-16	(58, 65)

aSLE, adult-onset SLE; cSLE, childhood-onset SLE; Anti-dsDNA, anti-double-stranded deoxyribonucleic acid; RNP, ribonucleic acid; aCL, anticardiolipin antibodies.

SLE cannot be cured, disease activity fluctuates with periods of flare and remission. Long-term maintenance of LLDAS is the goal of current treatment. Previous research has shown that cSLE is less likely to achieve LLDAS than aSLE is (7). Additionally, the specific physical and psychological characteristics of children and adolescents render cSLE patients more vulnerable to the long-term effects of the disease. Hydroxychloroquine and immunosuppressive drugs have improved the prognosis of SLE. The efficacy of “new” biologic agents, such as belimumab and telitacicept, is exciting. However, GC remains the mainstay of SLE. GCs are required by 97% and 70% of cSLE and aSLE patients, respectively, and their average doses are higher in children than in adults (73). cSLE patients not only have a higher Systemic Lupus International Collaborating Clinics/American College of Rheumatology (SLICC/ACR) Damage Index (SDI), but also accumulate damage faster (74), which is mainly related to GC toxicity, although time is considered. Given that GC play an irreplaceable role in the treatment of cSLE, their proper use is particularly important.

## 3 Glucocorticoids in cSLE: GC pulse therapy may be more advantageous

The intensity of GC is as follows: low dose ≤7.5mg/d prednisone equivalent; medium dose >7.5mg/d, but ≤30mg/d prednisone equivalent; high dose >30mg/d, but ≤ 100mg/d prednisone equivalent; pulse therapy ≥250mg/d for one or a few days (75). The use of high dose oral GC have become the rule for treating moderate to severe SLE activity (76). Recent data suggested that GC pulse therapy may have a biologic rational in the treatment of SLE, especially of cSLE.

### 3.1 Mechanism of GC action: genomic and non-genomic effects

**Figure 3:**
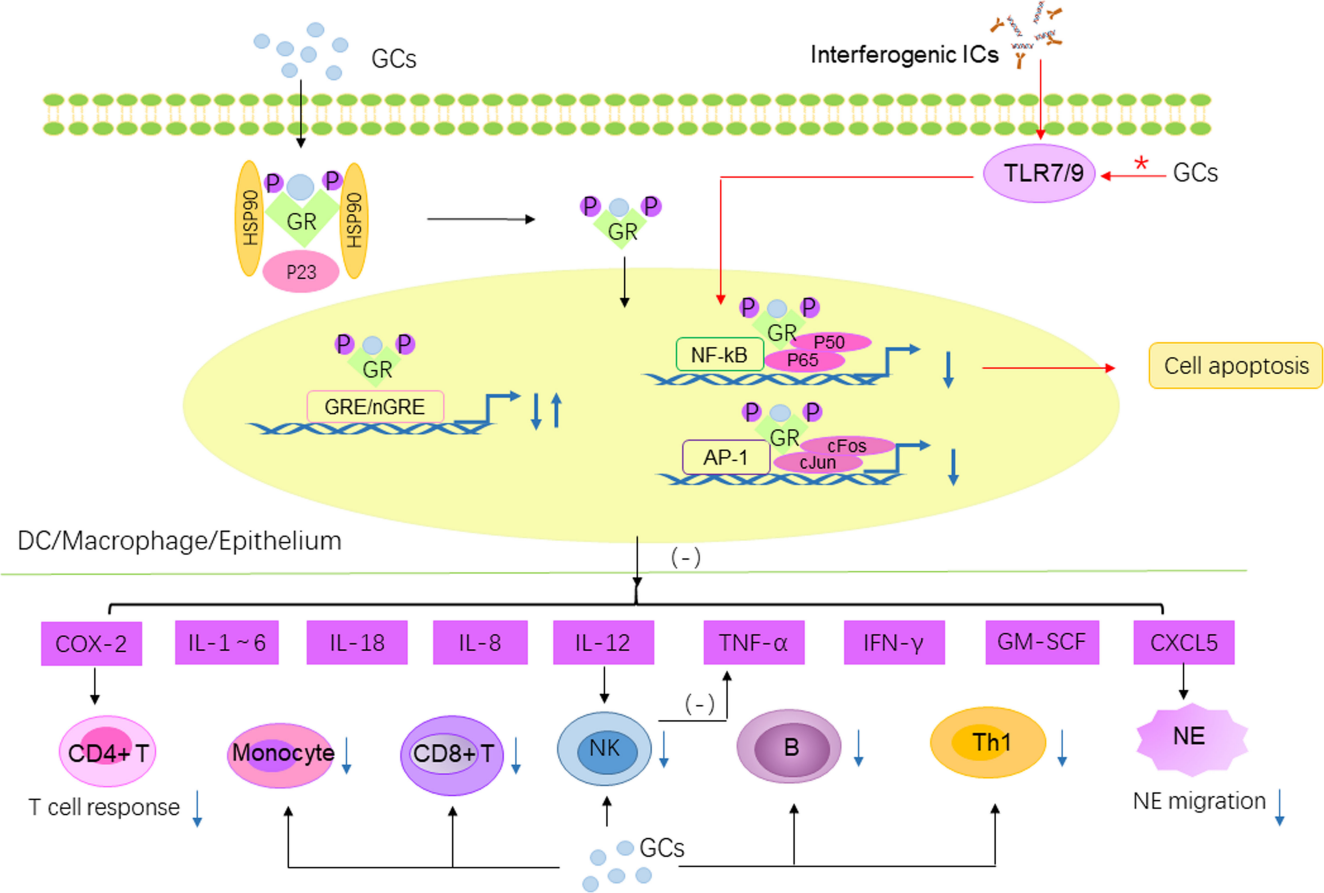
The anti-inflammatory/immunosuppressive effects of GC and difference between oral and pulsed GC. The effects of GC are transmitted intracellularly through the binding of GR to GRE/nGRE to suppress the expression of pro-inflammatory genes. Furthermore, GR can interact with p65 subunit of NF-κB to repress NF-κB regulated gene expression. GR can also interact with c-Jun to repress AP-1 regulated gene transcription. Then the synthesis of IL-1, IL-2, IL-3, IL-4, IL-5, IL-6, IL-8, IL-12, IL-18, GM-CSF, TNF-α, IFN-γ are decreased. The decreased IL-12 further reduced the secretion of TNF-α by NK cells. GR negatively regulates CXCL5, which in turn decreases NE migration. On the other hand, GR suppress the excessive inflammatory response mediated by T cell response by inhibiting COX-2. Finally, GC directly decreased the number of multiple immune cells including NK, CD8^+^ T cells, B cells, Th1 cells and monocytes. Pathway marked by red asterisks and red arrows is the different pathway affected by oral and pulse GC. The GC pulse regimen affects TLR7/9 in the IFN pathway, thereby affecting the activation of NF-κB, which in turn mediates pDC apoptosis and reduces IFN-α production. IFN pathway is not significantly affected by oral GCs. GRE, glucocorticoid response elements; nGRE, negative GRE; GM-CSF, granulocyte macrophage-colony stimulating factor; NE, neutrophil; TNF-α, tumor necrosis factor-α.

The anti-inflammatory and immunosuppressive actions of GC are exerted by two different mechanisms. Classic genomic effects are mediated by cytosolic GC receptors (GR). GC diffuse through the plasma membrane into the cytoplasm, forming a multiprotein complex with GR (GC-GR). The activated GC-GR complex moves to the nucleus and binds to specific GC-responsive elements (GRE), activating the transcription of specific genes and the synthesis of specific regulator proteins; this process is known as transactivation (**Figure 3**). Alternately, the GC-GR complex inhibits the activity of transcription factors such as activator protein 1 (AP-1) or NF-κB through direct or indirect action, thereby reducing the production of proinflammatory factors (77); this process is known as transrepression (**Figure 3**). In most cases, the anti-inflammatory and immunomodulatory effects of GC are achieved through transrepression, and transactivation mediates its side effects. A prednisone dose of >100 mg/day led to complete saturation of cGR, and non-genomic effects were activated. Three main modes of action exist for non-genomic effects. The GC-GR complex directly blocks the activation of phospholipase A2, thereby inhibiting the release of arachidonic acid. In contrast, GC rapidly activate GR on cell membranes, mediating rapid signal transduction via p38 MAPK. In addition, GC can alter cellular function by influencing cation transport, which contributes to rapid immunosuppression (78). The clinical impacts of non-genomic effects are rapid and play an important role in mediating GC pulse therapy. In contrast, genomic effects are often not immediate and are the primary activation mechanism of daily oral GC therapy. The toxicity and anti-inflammatory effects of the genomic effect increase in parallel with the dose. However, activating non-genomic effects provides additional benefits to patients with no increased toxicity (79). Ruiz-Arruza et al. found that the mean daily prednisone dose was higher in patients accruing GC-attributable damage (11 vs 7 mg/day, *p*=0.04) and MP pulses are not associated with damage accrual, suggesting that the dose of MP pulse is not included in the cumulative dese, which may be attributed to its rapid metabolism, as it is administered for a short time rather than continuously every day. Therefore, high-dose GC pulse can fully exert non-genomic effects and exert anti-inflammatory effect more quickly and fully. This is critical for children with SLE who require long-term management, as reducing organ damage in the early stage of disease helps to improve prognosis.

### 3.2 Pharmacologic effects of GC

Almost all primary and secondary immune cells are target for GC effects. As mentioned above, GC act by binding to GR that stimulates or inhibits gene expression. The anti-inflammatory and immunosuppressive effects of GC include inhibiting the synthesis of inflammatory cytokines and inducing the apoptosis of immune cells (See **Figure 3** for details). Clinical effects of GC are strongly dependent on the solubility, the rate of absorption, the metabolic rate, the affinity of GC to its receptor and the dose administered. Prednisone is commonly used as oral therapy, whereas methylprednisolone (MP) is often used as pulse therapy for its high bioavailability and wide distribution. In general, more than 90% of circulating GC is bound to transcortin (80). When transcortin is saturated, GC bind to albumin or remain unbound. Prednisone shows nonlinear protein binding, whereas MP is strictly linear, possibly because it binds albumin and not transcortin (81). In terms of GC absorption, although GC have a high oral absorption rate and bioavailability of > 60%, intravenous administration promotes a more rapid onset of action and higher bioavailability (82). And what’s more, compared with adults, the total body clearance (CL) of MP is often higher in children administered high-dose MP pulse therapy, indicating that individual children are more tolerant to MP pulse therapy (83).

### 3.3 Effects of GC on type I IFN

Although GC pulse therapy is considered to have lesser systemic side effects, there are few studies in literature comparing the effects of GC pulse therapy versus daily oral GC on the pathogenesis of SLE. Guiducci et al. (84) found that the daily oral prednisone returned to normal multiple transcriptional modules, except for the IFN pathway. In contrast, intravenous MP (IVMP) pulse therapy normalized the IFN signature. Consistent with this, the number of pDCs in the IVMP pulse group was significantly reduced, accompanied by decreased IFN-α levels (**Figure 3**). In this study, further experiments confirmed that stimulation of pDCs though TLR7/9 can account for the reduced activity of GC to inhibit the IFN pathway in SLE. And activation of NF-κB is crucial for the survival of pDCs. Interestingly, recognition of self-nucleic acid by TLR7/9 is an important step in the pathogenesis of cSLE, promoting the production of IFN-I. Combined with the overexpression of IFN-regulated genes and TLRs are more closely related to cSLE (46, 56), suggesting that IVMP pulse therapy may be more suitable for cSLE.

### 3.4 GC-related side effects

Children are more susceptible to the side effects of GC than adults, especially regarding growth, development, mental and psychological aspects. GC-related toxicity is highly dependent on the time and dose of exposure. A safe dose has not been established, and GC-related damage occurs even at doses of 4.5-7.5mg/day (85). The effect of GC on bone is worth noting, as it is closely related to osteoporosis and osteonecrosis. The persistent use of GC in children can lead to delayed puberty and short stature in adulthood (86). This may be related to the GC-induced inhibition of the hypothalamic-pituitary-adrenal (HPA) axis and the decrease in bone mineral density. Some studies have demonstrated that GC pulse therapy does not lead to a decrease in bone mineral density and is not independently correlated with osteonecrosis (87). Additionally, a study on multiple sclerosis (MS) using GC pulse therapy found no prolonged suppressive effect on the HPA axis (88). This might explain the reduced severity of Cushing’s syndrome in children treated with GC pulse therapy compared with those treated with daily oral therapy (89). Neuropsychiatric symptoms are another common adverse effect of GC in children. Epileptic seizures, behavioral abnormalities, and cognitive impairment are the most common neuropsychiatric manifestations in cSLE patients treated with GC. However, there is evidence that neuropsychiatric symptoms are not associated with GC pulse therapy (90). In addition to organ damage caused by persistent disease activity, GC is an independent risk factor for cSLE damage. And studies on aSLE have shown that GC pulse therapy is not associated with damage accrual. Infection and hyperglycemia are other common adverse reactions. Although fasting blood glucose (FBG) levels are elevated during GC pulse therapy, FBG levels in non-diabetic patients slowly return to normal after the pulse ends (90). Major infections can occurre at a median oral prednisone dose of only 7.5mg, and the risk of infection increased 11-fold for every 10mg daily increase in prednisone dose (91). Infection appears to be the side effect associated with GC pulse therapy that requires special attention. However, some researchers suggest that GC pulses have no independent effect on infections (92).

**Figure 4:**
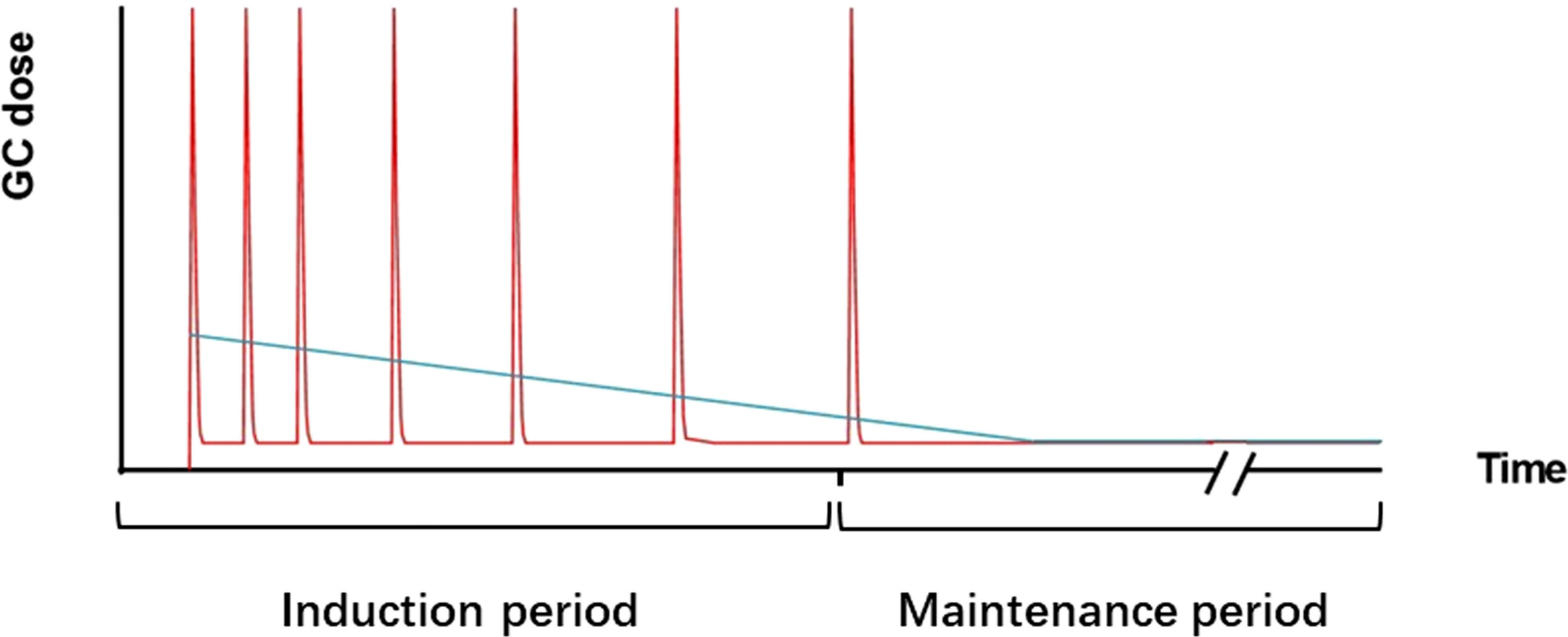
Pattern-trend plot of GC therapy. The red line represents the repeated IVMP pulses combined with low-dose oral prednisone therapy. The blue line represents the daily oral prednisone therapy. In daily oral therapy, prednisone doses decreased gradually but mostly remained at higher levels. In intermittent IVMP pulses combined with low-dose oral prednisone therapy, MP doses were high during the pulses, and intermittent and maintenance periods were given a low dose of GCs.

GC pulses were first used for the treatment of SLE in the 1970s and have been used ever since. An observational cohort study showed that IVMP pulse therapy reduced the subsequent oral doses of prednisone (93). The use of reduced oral prednisone doses decreased GC-related damage and improved cardiovascular prognosis without increasing SLE-induced damage (94). These findings suggest that IVMP pulse therapy is superior to high-dose daily oral prednisone therapy. The Lupus-Cruces protocol, based on the above evidence, has attracted much attention in recent years. This protocol can be summarized as intermittent IVMP pulses combined with low-dose oral prednisone. Compared to traditional GC treatment regimens, intermittent IVMP pulses reduce the dose of oral prednisone and enhance the clinical response (95). A recent observational cohort study showed a higher rate of prolonged remission in the Lupus-Cruces group (96). All of the above suggested that intermittent IVMP pulse combined with low-dose oral prednisone have good application prospects for the treatment of SLE. Details of this GC administration pattern are provided in **Figure 4**. It should be noted that there is significant variation in individual patient responsiveness to GC therapy, especially in patients with associated immunodeficiency. Agents targeting genes and genetic pathways are currently under more intensive investigation for patients with immunodeficiency in cSLE (97).

## 4 Summary

cSLE has more severe clinical manifestations and injuries earlier than aSLE does. Compared to adult-onset SLE, cSLE has a stronger genetic background and more prevalent elevated IFN-I expression. Guidelines and consensus recommendations for moderate to severe cSLE are high-dose daily oral GC. In view of the side effects and irreplaceability of GC, the key approach for a reasonable and successful systematic GC treatment is to minimize the dose of the administered GC in order to reduce the occurrence of side effects. Of course, immunosuppressive agents are necessary regardless of GC application regimen. GC pulse therapy can rapidly exert anti-inflammatory and immunosuppressive effects with reduced GC-related toxicity and allowing a reduction in oral prednisone doses. Combined with the pharmacology, activation mechanism and the specific effect on IFN of the GC pulse, intermittent IVMP pulse therapy may be the preferred treatment for cSLE rather than the current daily oral therapy. Further clinical and experimental studies are required to support this idea.

## Author contributions

All authors listed have made a substantial, direct, and intellectual contribution to the work, and approved it for publication.
